# Non-attendance at psychiatric outpatient clinics: comparison of clinical, risk and demographic factors between attenders and non-attenders

**DOI:** 10.1192/bjo.2021.145

**Published:** 2021-06-18

**Authors:** Mahum Kiani, Nilamadhab Kar

**Affiliations:** Black Country Healthcare NHS Foundation Trust

## Abstract

**Aims:**

With an overarching aim of decreasing the incidence of non-attendance in psychiatric outpatient clinics, this service evaluation was intended to explore the profile of non-attenders. Specifically, the clinical, risk and demographic features of patients who did not attend their psychiatric outpatient appointments were compared with those of attenders. The outcome of patients who did not attend was also studied.

**Method:**

All the consecutive non-attenders (n = 32) in November 2020 in a psychiatric outpatient clinic were compared with 32 consecutive attenders. The groups were compared based on clinical features (diagnosis, medical treatment, psychological treatment, care programme approach, first contact), risk profile (self or others) and demographic features (age, gender, ethnicity, accommodation, occupation, benefits). The non-attender sample was also analysed to consider the outcome after their missed appointment, following local Trust protocols.

**Result:**

The overall rate of patients who did not attend their appointment was 22%. There was a statistically significant difference between the age and gender of non-attenders. Males were less likely to attend their appointment than females (p = 0.024). The mean age of patients who did not attend their appointment was 36.4 compared with 44.8 years in the attenders (p = 0.005). There were a few clinically relevant findings. Around one third (34%) of patients who did not attend their appointments had a history of risk of self-harm noted in previous appointments. The results also showed that 75% of individuals who did not attend their outpatient appointments were unemployed. There were no significant differences based on the type of treatments (depot injections, lithium, clozapine, antipsychotics or antidepressants) patients received. Patients who did not attend were more likely to have a mood disorder (59% compared with 40%), and less likely to have a psychotic disorder (25% compared with 44%). Of the patients who did not attend, all were appropriately contacted as per the local Trust guidelines via a letter, and were provided with appointments where appropriate; 34% of non-attenders were discharged from services.

**Conclusion:**

Non-attendance at psychiatric outpatient appointments is a concern, particularly for younger and male patients. Considering the clinical risks associated with this patient population, efforts need to be taken to improve their engagement with mental health services. Future studies may explore patients’ perspectives of non-attendance and how to ameliorate any hindrances to attending.

